# An audit of lithium prescribing practices in an old age psychiatry service highlighting renal impairment in this cohort

**DOI:** 10.1192/bjo.2021.323

**Published:** 2021-06-18

**Authors:** Leia Valentine, John Cannon, Siobhan Marmion, Michelle Corcoran, Marguerite Cryan, Geraldine McCarthy, Catherine Dolan

**Affiliations:** Liscarney House, Psychiatry of Old Age Service

## Abstract

**Aims:**

To compare Lithium prescribing practices in a Psychiatry of Old Age (POA) Service in the North-West of Ireland among adults aged 65 years and over with best practice guidelines.

**Method:**

Review of the literature informed development of audit standards for Lithium prescribing. These included National Institute for Clinical Excellent (NICE) 2014 guidelines, The British National Formulary (2019) and Maudsley Prescribing Guidelines (2018). Data were collected retrospectively, using an audit-specific data collection tool, from clinical files of POA team caseload, aged 65 years or more and prescribed Lithium over the past one year.

**Result:**

At the time of the audit in February 2020, 18 patients were prescribed lithium, 67% female, average age 74.6 years. Of those prescribed Lithium; 50% (n = 9) had a depression diagnosis, 44% (n = 8) had bipolar affective disorder (BPAD) and 6% (n = 1) had schizoaffective disorder.

78% (n = 14) of patients were on track to meet, or had already met, the NICE standard of 3-monthly serum lithium level. Lithium levels were checked on average 4.5 times in past one year, average lithium level was 0.61mmol/L across the group and 39% (n = 7) had lithium level within recommended therapeutic range (0.6-0.8mmol/L).

83% (n = 15) of patients met the NICE standards of 3 monthly renal tests, thyroid function test was performed in 89% (n = 16) and at least one serum calcium level was documented in 63% (n = 15). Taking into consideration most recent blood test results, 100% (n = 18) had abnormal renal function, 78% (n = 7) had abnormal thyroid function and 60% (n = 9) had abnormal serum calcium.

Half (n = 9) were initiated on lithium by POA service and of these, 56% (n = 5) had documented renal impairment prior to initiation. Of patients on long term lithium therapy at time of referral (n = 9), almost half (n = 4) had a documented history of lithium toxicity.

**Conclusion:**

The results of this audit highlight room for improvement in lithium monitoring of older adults attending POA service. Furthermore, all patients prescribed lithium had impaired renal function, half had abnormal calcium and two fifths had abnormal thyroid function. This is an important finding given the associations between those admitted to hospital with COVID-19 and comorbid kidney disease and increased risk of inpatient death.

Our findings highlight the need for three monthly renal function monitoring in older adults prescribed lithium given the additive adverse effects of increasing age and lithium on the kidney. Close working with specialised renal services to provide timely advice on renal management for those with renal impairment prescribed lithium is important to minimise adverse patient outcomes.

